# Skin Hydration Effects of Scale-Up Fermented *Cyclopia intermedia* against Ultraviolet B-Induced Damage in Keratinocyte Cells and Hairless Mice

**DOI:** 10.1155/2020/3121936

**Published:** 2020-01-11

**Authors:** A-Rang Im, Sung Hum Yeon, Kon-Young Ji, Rak Ho Son, Key An Um, Sungwook Chae

**Affiliations:** ^1^Herbal Medicine Research Division, Korea Institute of Oriental Medicine, 1672 Yuseong-daero, Yuseong-gu, Daejeon 34054, Republic of Korea; ^2^Biomodulation Major, Department of Agricultural Biotechnology, Seoul National University, Seoul 157-742, Republic of Korea; ^3^Huons Co., Ltd., College of Pharmacy, Hanyang University, Ansan 426-791, Republic of Korea; ^4^Korean Convergence Medicine, University of Science and Technology, 217 Gajeong-ro, Yuseong-gu, Daejeon 34113, Republic of Korea

## Abstract

Photoaging occurs by chronic skin exposure to the sun and ultraviolet irradiation and leads to skin aging accompanied by a lack of skin hydration. We previously demonstrated the photoprotective effect of fermented *Cyclopia intermedia* (honeybush) extract on the skin. In this study, we evaluated the skin hydration effects of scaled-up fermented honeybush extract (HU-018) against ultraviolet B (UVB) radiation in HaCaT immortalized human keratinocytes and hairless mice. Pretreating HaCaT cells with HU-018 attenuated the decreased hyaluronic acid (HA) levels and mRNA expression of genes encoding involucrin, filaggrin, and loricrin by UVB irradiation. HU-018 treatment also ameliorated the decreased stratum corneum (SC) hydration and the increased levels of transepidermal water loss (TEWL) and erythema index (EI) in hairless mice after UVB exposure. Microarray analysis revealed changes in gene expression patterns of hyaluronan synthase 2 (Has2), transforming growth factor-beta 3 (TGF-*β*3), and elastin induced by HU-018 in UVB-irradiated mice. Consistently, the mRNA expression of Has2, TGF-*β*3, and elastin was increased by HU-018 treatment. Moreover, HU-018 restored the increased epidermal thickness and collagen disorganization in skin tissue of UVB-irradiated mice. HU-018 treatment also decreased matrix metalloproteinase-1 (MMP-1) expression and increased procollagen type-1, elastin, and TGF-*β*1 expression. In conclusion, we found that HU-018 promoted skin hydration processes in UVB-irradiated keratinocytes and hairless mice by modulating involucrin, filaggrin, loricrin, and HA expression and ameliorating visible signs of photoaging. Thus, HU-018 may be a good skin hydration agent for skin care.

## 1. Introduction

*Cyclopia intermedia* (honeybush) is a herbal tea indigenous to South Africa that is traditionally used for medicinal purposes and is highly similar to Rooibos [1]. Honeybush is rich in polyphenols and is a rare source of the dietary dihydrochalcones aspalathin and nothofagin [2]. Aqueous extracts of honeybush have been reported to have antimutagenic activities against 2-acetyl laminofluorence- and aflatoxin B1-induced mutagenesis and chemoprotective properties against cancer [3–5].

In a previous study, we presented evidence of the antiwrinkle activity of fermented *C. intermedia* (honeybush) extract and demonstrated the feasibility of using this extract in animal models [6]. However, the production of fermented honeybush extract would need to be scaled-up for use in a clinical trial, both in terms of quantity and cost. Normally, basic laboratory-scale studies are designed to determine the *in vivo* efficacy of an active pharmaceutical ingredient in the early stages, without specific regard to its safety, production cost, or stability of the development process of the product. However, transitioning from laboratory-based research to the trial phase requires scaling-up the production of the active ingredient to establish its safety and efficacy, as well as to ensure cost-effective production. For the use of fermented honeybush extract in clinical trials, we modified the process to yield scaled-up fermented honeybush extract (HU-018), after confirming the nontoxicity of HU-018 in Sprague Dawley rats and beagles, and confirmed that HU-018 met the requirements for commercialization as an antiaging agent. In addition, the effects of HU-018 on UVB-irradiated damage were previously evaluated in HaCaT cells [7].

Aging of the human skin is a complex biological process that occurs due to a combination of endogenous (intrinsic) and exogenous (extrinsic) factors [8]. Environmental factors including ultraviolet (UV) exposure, alcohol intake, pollution, and severe physical stress result in the development of extrinsic aging [9]. Ultraviolet B (UVB) exposure is the most important extrinsic factor that accelerates skin aging, a process that is commonly termed photoaging [10]. Skin aging is characterized by the loss of elastic and collagen fiber network, due to the presence of dysfunctional fibroblasts, with the loss of structure leading to wrinkle formation [11]. In photoaged skin, dermal changes are observed, such as a reduction in the amount of collagen and precursors of type I and III collagens, as well as a degeneration of elastic fibers [12].

The skin is important for protecting the body against dehydration and environmental factors including temperature, variations in humidity, and sun exposure [13]. UVB radiation alters epidermal morphology by increasing the thickness of the stratum corneum (SC), which causes an imbalance in the permeability of the SC barrier, and thus increases transepidermal water loss (TEWL) [14].

One of the most important indicators of skin barrier function in the cosmetic and skin pathology field is skin hydration [15]. Skin aging is also associated with skin water loss, the main factor being hyaluronic acid (HA), an extracellular matrix molecule [16]. Several factors control skin moisture and elasticity, including HA and elastic fibers, which regulate skin tissue elasticity and resilience [17]. Enzymes such as HA synthases (Has) synthesize HA, and Has2 expression is differentially upregulated by TGF-*β*1 in the dermis and epidermis [18]. Additionally, involucrin, filaggrin, and loricrin play important roles to promote terminal differentiation of the epidermis and formation of the skin barrier [19].

Based on the evidence mentioned above suggesting the feasibility of using fermented honeybush extract for inhibiting photoaging-induced skin hydration, our aim in this study was to evaluate the skin hydration effects of the scale-up fermented HU-018 in a HaCaT cells and a hairless mouse model.

## 2. Materials and Methods

### 2.1. Preparation of HU-018

HU-018 was prepared as per a previously described method [7] with slight modifications. In brief, honeybush (*Cyclopia intermedia*) was obtained from Rooibos Ltd. (Clanwilliam, South Africa). To prepare the honeybush extract, raw material was extracted twice with water for 1 h under reflux conditions. The extract was filtered and then evaporated to yield 20 brix (the solid content is about 20% (20 g/100 g)). A lactic acid bacterium (*Streptococcus thermophilus*) was obtained from Lallemand Health Solutions, Inc (Montreal, Canada). To prepare the fermented honeybush extract, 10% of lactic acid bacterium (*S. thermophilus*, 1 × 10^5^ cfu/ml) was inoculated in 0.5% whole milk powder (Seoulmilk Co., Seoul, Korea) with 3% lactose (Sung Poong Co. Ltd., Seoul, Korea), 23% honeybush extract (20 brix), and 63.5% purified water. The mixture was fermented in an incubator at 37°C for 48 h. After sterilizing the fermented mixture, the final product (HU-018) was obtained by mixing with dextrin (DE12; Roquette, Lestrem, France) and spray-drying.

### 2.2. Cell Culture and UVB Irradiation

The detailed methods have been described previously [7]. In brief, HaCaT cells were purchased from CLS Cell line service (Eppelheim, Germany) and maintained in Huons Co., Ltd. (Gyeonggi-do, Korea). We obtained the HaCaT cells from Huons, and the cells were cultured in Dulbecco's modified Eagle medium (DMEM; Gibco, Rockville, MD, USA) containing 10% heat-inactivated fetal bovine serum (FBS, Gibco) and 1% antibiotics (Gibco) at 37°C and 5% CO_2_ in a humidified incubator. The cells were plated and allowed to adhere for 24 h, and then treated with various concentrations of HU-018. After 24 h, the medium was changed and exposed to UVB radiation at a dose of 20 mJ/cm^2^. UVB irradiation was performed using a UVM-225D Mineralight UV display lamp (UVP, Phoenix, AZ, USA) that emitted at a wavelength of 302 nm. The strength of the UV radiation was measured using a HD2102-2 UV meter (Delta OHM, Padova, Italy).

### 2.3. Experimental Animals and Oral Administration

All animal experiments were approved by the Institutional Animal Care and Use Committee of Kyunghee University (approval number: KHUASP(SE)-16-008). The experimental protocols and animal care were carried out following the National Research Council Guide for the Care and Use of Laboratory Animals. Female HR-1 hairless mice (5 weeks old) were purchased from Dae Han Bio Link Co., Ltd. (Chungcheongbuk-do, Korea) and allowed to habituate to the laboratory for 1 week before the animal experiments. The hairless mice were housed in a controlled environment (23 ± 3°C at 55 ± 15% humidity) with a 12 : 12-h light/dark cycle. The mice were given free access to water and food. For the experimental evaluation, the mice were divided into 7 groups, with 10 animals in each group: the normal group, UVB-irradiated group treated with vehicle, UVB-irradiated group treated with HA (50 mg/kg), UVB-irradiated group treated with collagen (400 mg/kg), UVB-irradiated group treated with fingerroot (160 mg/kg), UVB-irradiated group treated with HU-018 at a low (80 mg/kg) concentration, and UVB-irradiated group treated with HU-018 at a high (160 mg/kg) concentration. Mice in the positive control (hyaluronic acid, collagen, and fingerroot) and HU-018 groups orally received 0.2 mL of 0.5% carboxymethyl cellulose daily for 12 weeks. Mice in the normal group and UVB-irradiated vehicle group were orally administered with 0.5% carboxymethyl cellulose for 12 weeks. The mice were sacrificed under anesthesia by using a mixture of tiletamine/zolazepam (Virbac, Carros, France) and xylazine (Bayer Korea Ltd., Seoul Korea) through intraperitoneal injection.

### 2.4. UVB Irradiation

The method for UVB irradiation was based on previously reported methods with slight modifications [20]. In brief, five Sankyo Denki sunlamps were used to perform the UVB irradiation with a peak irradiance at 310 nm (G9T5E lamps, Sankyo Denki, Hiratsuka, Japan). The UV source was positioned 15 cm above the backs of hairless mice. Irradiance was measured using an IL1700 Research Radiometer equipped with a UVB sensor (International Light, Inc., Newburyport, MA, USA). The mice were exposed to 100 mJ/cm^2^ UVB radiation (one minimal erythemal dose = 100 mJ/cm^2^) every day for the first week and then 200 mJ/cm^2^ UVB radiation every other day for 2 weeks. Then, mice were exposed to 400 mJ/cm^2^ UVB radiation twice a week for 2 weeks, and finally 100 mJ/cm^2^ UVB radiation every other day for 7 weeks.

### 2.5. Determination of HA Expression by ELISA

The expression of HA in the skin tissue of UVB-irradiated mice was evaluated using the total HA enzyme-linked immunosorbent assay (ELISA) kits according to the manufacturer's instructions (R&D Systems, Minneapolis, MN, USA). The expression level of HA was measured and quantified using a microplate reader (Molecular Devices, Sunnyvale, CA, USA).

### 2.6. Histological Examination

Dorsal skin specimens were obtained from hairless mice after final UVB irradiation, and the skin specimens were fixed using 4% paraformaldehyde for 24 h. After fixation, the specimens of dorsal skin were embedded in paraffin and sectioned at 5 *μ*m thickness. Epidermal thickness of skin specimens was analyzed after staining by hematoxylin and eosin (H&E), and collagen fibers were detected using Masson's trichrome staining. The stained skin specimens were analyzed under a microscope (Observer D2, Zeiss, Munich, Germany).

### 2.7. Physiological Analysis of Skin Functions

TEWL, SC hydration, and erythema index (EI) were evaluated using the relevant probes (DermaLab®; Combo, Cortex Technology). In this study, TEWL, SC hydration and EI were analyzed based on data obtained before exposure and after 12 weeks of UVB irradiation.

### 2.8. Preparation of Microarray Library and Sequencing

The microarray library was prepared and sequenced according to previously reported methods [21]. In brief, total RNA was extracted using Trizol reagent (Invitrogen, Carlsbad, CA, USA), and RNA quality was measured using an Agilent 2100 bioanalyzer and the RNA 6000 Nano Chip (Agilent Technologies, Amstelveen, The Netherlands). RNA was quantified using an ND-2000 spectrophotometer (Thermo Inc., DE, USA). A QuantSeq 3′ mRNA-Seq Library Prep Kit (Lexogen, Inc., Austria) was used to analyze the microarray library. The libraries were constructed according to the manufacturer's protocols. In brief, reverse transcription was performed after hybridizing each total RNA (500 ng) with an oligo-dT primer containing an illumina-compatible sequence at its 5′ end. The second strand was synthesized using a random primer containing an illumina-compatible linker sequence at its 5′ end, after degradation of the RNA template. All reaction components were incubated with magnetic beads to purify the double-stranded library. To add the complete adapter sequences required for cluster generation, the library was amplified. High-throughput sequencing was performed as single-end 75 sequencing by a NextSeq 500 (Illumina, Inc., USA) after purifying PCR components from the finished library.

### 2.9. Analysis of Microarray Data

Microarray data were analyzed according to previously reported methods [21]. In brief, QuantSeq 3′ mRNA-Seq reads were aligned using Bowtie2 [22]. Bowtie2 indices were either generated from the genome assembly sequence or the representative transcript sequences from aligning to the genome and transcriptome. The alignment file was used for assembling transcripts, estimating their abundances, and detecting differential gene expression. Differentially expressed genes were determined based on counts from unique and multiple alignments using coverage in Bedtools [23]. The RC (read count) data were processed based on global normalization method using Genowiz™ version 4.0.5.6 (Ocimum Biosolutions, India). Genes were classified based on searches in DAVID (http://david.abcc.ncifcrf.gov/) and Medline databases (http://www.ncbi.nlm.nih.gov/).

### 2.10. Analysis of mRNA Expression

The total RNA from HaCaT cells and skin of UVB-irradiated hairless mice was isolated using TRIzol reagent (Invitrogen) according to the manufacturer's instructions. mRNA expression was analyzed by quantitative real-time polymerase chain reaction (qRT-PCR) using TaqMan assays (Applied Biosystems, Foster City, CA, USA) and a QuantStudio^TM^ 6 Flex real-time PCR system (Applied Biosystems) according to the manufacturer's instructions. Each sample was assayed in triplicate. The mRNA levels were normalized to that of *β*-actin and calculated using the ^ΔΔ^Ct method.

### 2.11. Western Blotting Analysis

After the animals were sacrificed, all dorsal skin tissue samples were homogenized with lysis buffer (50 mM Tris-Cl, pH 8.0, 0.1% SDS, 150 mM of NaCl, 1% NP-40, 0.02% sodium azide, 0.5% sodium deoxycholate, 100 *μ*g/mL phenylmethanesulfonyl fluoride, 1 *μ*g/mL of aprotinin, and a phosphatase inhibitor), and then they were centrifuged at 12,000 ×g for 20 min. The concentration of protein lysates was determined using the Bradford reagent (Bio-Rad) using bovine serum albumin as a standard. Equal concentrations of protein lysates were separated through 8% or 10% SDS-polyacrylamide gel electrophoresis and transferred to a nitrocellulose membrane (Amersham Biosciences, Little Chalfont, UK). The membrane was incubated for 1 h with blocking buffer (5% nonfat milk in tris-buffered saline solution with Tween® (TBS-T)) at room temperature (RT). After blocking, the membranes were incubated with primary antibodies against MMP-1, procollagen type-1, elastin, TGF-*β*1, or *β*-actin (Santa Cruz Biotechnology Inc.) at 4°C overnight. After washing three times with TBS-T, the membranes were incubated for 1 h with secondary antibodies conjugated with horseradish peroxidase (HRP) (Santa Cruz Biotechnology Inc.) at R.T. Finally, the membranes were developed using an enhanced chemiluminescence (ECL) detection system (Amersham Biosciences) and an LAS-4000 image analyzer (Fujifilm, Tokyo, Japan).

### 2.12. Statistical Analysis

The data were analyzed using the Statistical Analysis System (GraphPad Prism 5, GraphPad Software, Inc., San Diego, CA, USA). All experiments were performed at least three times independently, and the data were calculated as means. The data are represented as mean ± standard deviation (SD). Statistical comparisons among groups were analyzed by one-way analysis of variance (ANOVA) using Tukey's multiple comparison test. *p* values <0.05 were considered statistically significant.

## 3. Results

### 3.1. Effects of HU-018 on Moisturizing-Related Genes and HA Levels in UVB-Irradiated HaCaT Cells

**Figure 1:**
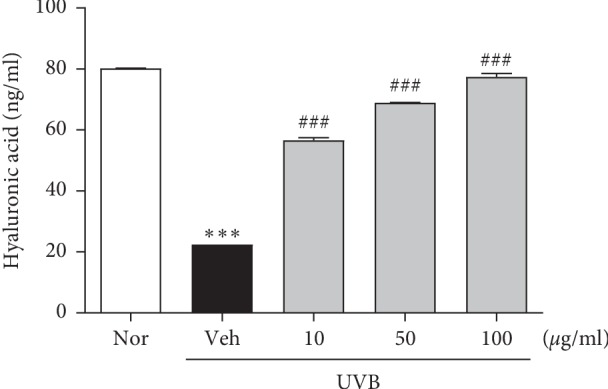
Effects of HU-018 treatment on hyaluronic acid expression in UVB-irradiated HaCaT cells. Hyaluronic acid secretion in HaCaT cells exposed to UVB. ^*∗∗∗*^*p* < 0.001 versus the normal group; ^###^*p* < 0.001 versus the UVB-irradiated vehicle group. Nor, non-irradiated group; veh, UVB-irradiated group.

In our previous study, we investigated the expression of involucrin, filaggrin, and loricrin in UVB-induced HaCaT cells after treatment with HU-018 [7]. Consistently, the mRNA expression of genes encoding involucrin, filaggrin, and loricrin decreased upon UVB exposure in HaCaT cells compared with expression in normal control cells, and their expression increased upon treatment with HU-018 (Supplementary [Supplementary-material supplementary-material-1]). ELISA analysis revealed that HA levels were markedly decreased in UVB-irradiated HaCaT cells and HU-018 treatment increased HA levels in a dose-dependent manner (Figure 1).

### 3.2. Evaluation of TEWL, SC Hydration, and EI

**Figure 2:**
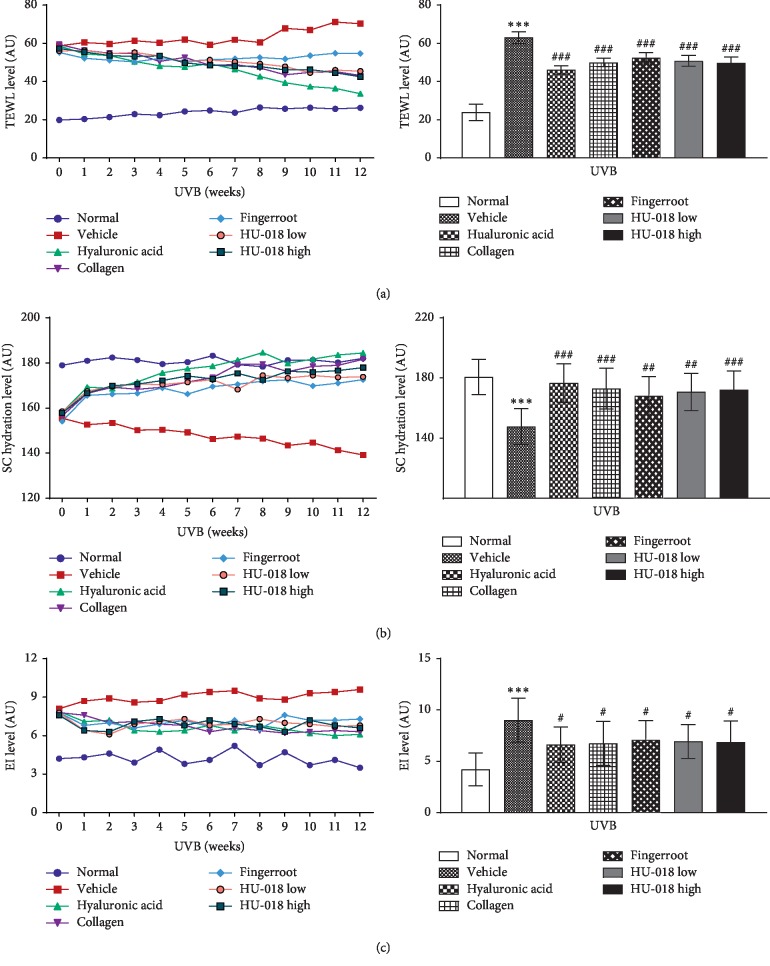
Evaluation of the effects of the 12-week UVB irradiation on (a) TEWL, (b) SC hydration, and (c) EI in the hairless mice model. ^*∗∗∗*^*p* < 0.001 versus the normal group, and ^#^*p* < 0.05, ^##^*p* < 0.01, and ^###^*p* < 0.001 versus the UVB-irradiated vehicle group. SC, stratum corneum; TEWL, transepidermal water loss; EI, erythema index; UVB, ultraviolet B; HU-018, scaled-up fermented honeybush extract. Normal, non-irradiated group; vehicle, UVB-irradiated group.

There was no difference in the mortality rate of mice across the 7 experimental groups for 12 weeks. Moreover, the body weight was comparable across the 7 groups (data not shown). After UVB irradiation, the hydration level decreased in the SC, with a concomitant increase in TEWL and EI of hairless mice skin (Figure 2). HU-018 treatment attenuated the effects of UVB irradiation on decreasing SC hydration, TEWL, and EI levels to levels similar or better to those observed in the positive controls (HA, collagen, and fingerroot).

### 3.3. Microarray Analysis

**Figure 3:**
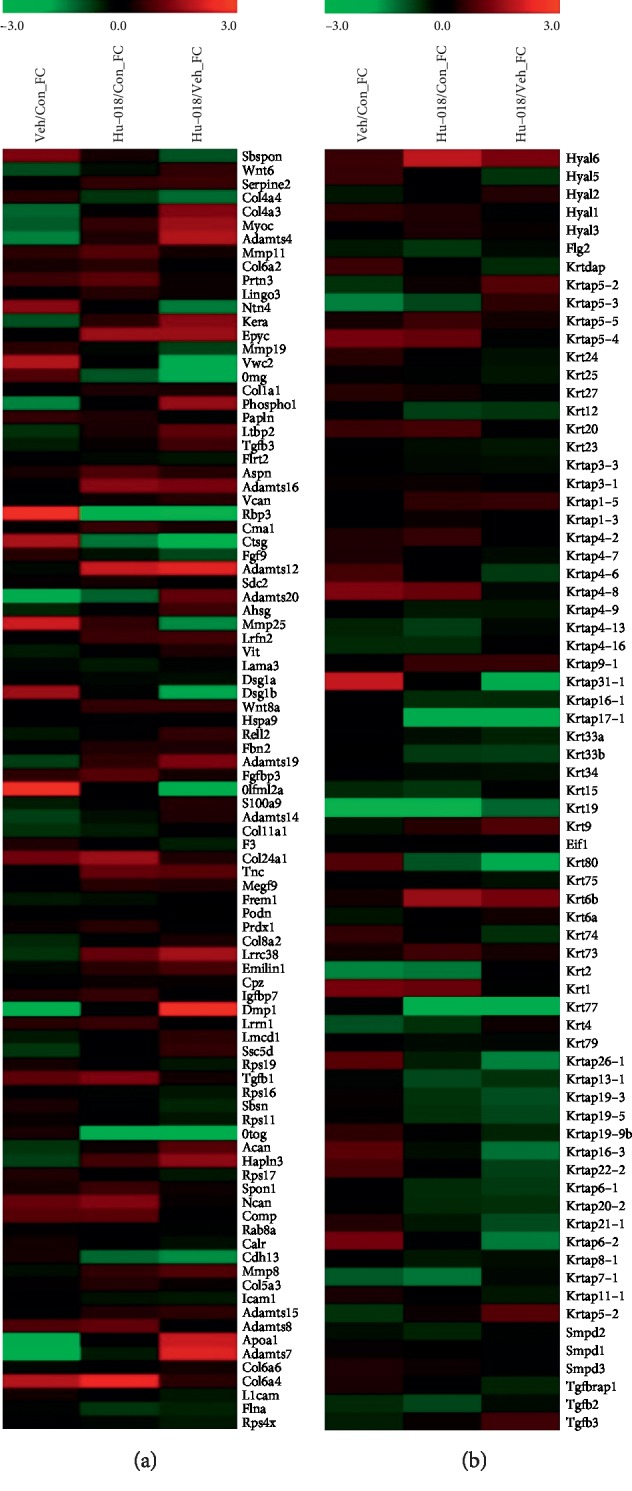
Expression profiles of genes regulated by HU-018 in UVB-irradiated hairless mice. These subsets of genes were clustered hierarchically based on the similarity of their expression profiles. (a) Genes of extracellular matrix and (b) skin hydration-related genes. For each gene, the ratio of mRNA levels in UVB-irradiated hairless mice after administration of HU-018 is represented by color, according to the color scales at the top of the figure.

To verify the microarray results, HU-018 treatment caused differential regulation of over 20,000 genes between the HU-018 administration animal group and the UVB-irradiated vehicle group. The expression of genes related to the extracellular matrix (ECM) was changed in the skin of hairless mice; 63 genes were upregulated, and 31 genes were downregulated (Figure 3(a)). Among the genes related to skin hydration, 20 genes were upregulated and 51 genes were downregulated (Figure 3(b)). Cluster analysis showed that UVB irradiation downregulated the expression of skin hydration-related genes including COL1A1, Has-2, TGF-*β*3, and elastin, and expression of these genes was upregulated by HU-018 administration. Moreover, HU-018 administration normalized the gene expressions of proteoglycans such as Acan, Kera, and Podn which were downregulated by UVB irradiation. HU-018 administration also downregulated the gene expression of keratin-1 and keratin-associated proteins, whose expression is well known to protect epithelial cells from damage or stress.

### 3.4. Effect of HU-018 on Skin Hydration Factors

**Figure 4:**
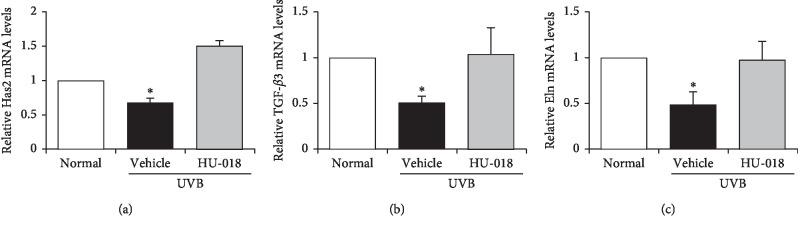
Effects of HU-018 on Has2, TGF-*β*3, and elastin expression in UVB-irradiated hairless mice. Expression of (a) Has2, (b) TGF-*β*3, and (c) elastin mRNA was determined by qRT-PCR. ^*∗*^*p* < 0.05 versus the normal group. HU-018, scaled-up fermented honeybush extract; UVB, ultraviolet B; mRNA, messenger RNA; qRT-PCR, quantitative real-time polymerase chain reaction. Normal, non-irradiated group; vehicle, UVB-irradiated group.

To confirm the microarray findings, mRNA expression of skin hydration factors was analyzed by qRT-PCR in skin tissue of UVB-irradiated hairless mice. The qRT-PCR results showed that UVB irradiation suppressed the expression of genes encoding Has2, TGF-*β*3, and elastin (Figure 4), and these changes in expression reversed by HU-018 treatment in UVB-irradiated mice.

### 3.5. Evaluation of Antiwrinkle Effects by Histological Staining

**Figure 5:**
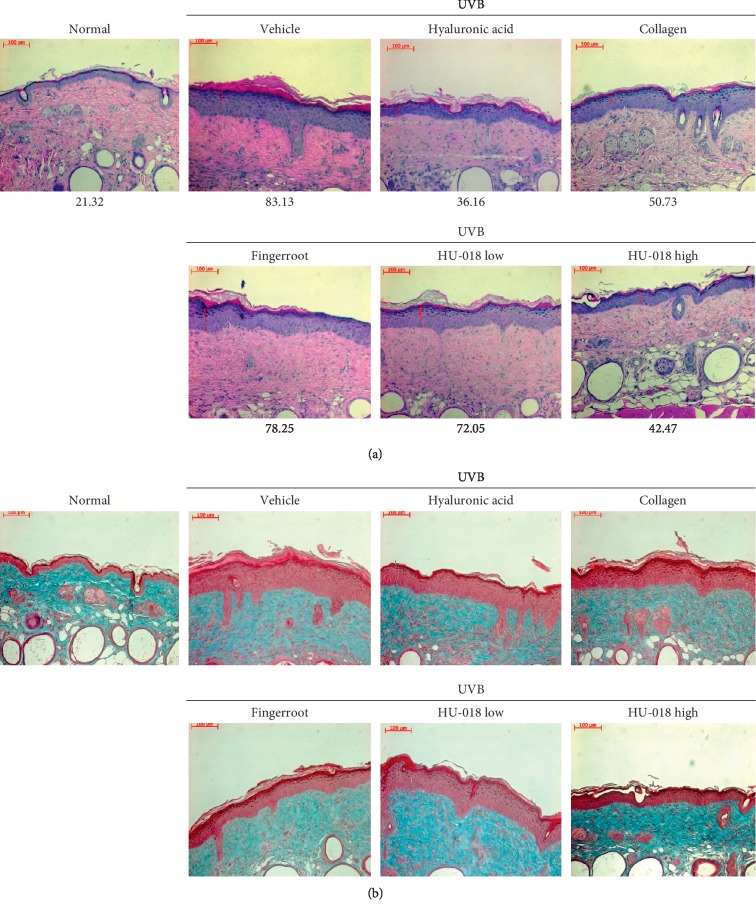
Effect of HU-018 on UVB irradiation-induced epidermal thickening in the dorsal skin of hairless mice. (a) Hematoxylin and eosin-stained images of UVB-irradiated dorsal skin of mice. The number (*μ*m) is the epidermal thickness. (b) Protective effects of HU-018 on photoaging of the skin regarding changes in collagen fiber volume determined using Masson's trichrome staining. Collagen fibers are stained in blue, and images were obtained under 200x magnification. Scale bar = 100 *μ*m. HU-018, scaled-up fermented honeybush extract; UVB, ultraviolet B. Normal, non-irradiated group; vehicle, UVB-irradiated group.

To evaluate the wrinkle alleviation effect of HU-018, skin tissue samples from hairless mice were analyzed after staining with H&E and Masson's trichrome. The H&E-stained skin tissue showed that the thickness of the SC and epidermis was significantly higher in the UVB-irradiated vehicle group than that in the normal group (Figure 5(a)). However, HU-018 treatment resulted in similar or better SC and epidermal thickness values as those in the positive control groups. Moreover, a greater volume of collagen fibers was observed upon HU-018 treatment than was observed in the vehicle group after Masson's trichrome staining (Figure 5(b)).

### 3.6. Effect of HU-018 on Procollagen Type-1, Elastin, and TGF-*β*1 Expression

**Figure 6:**
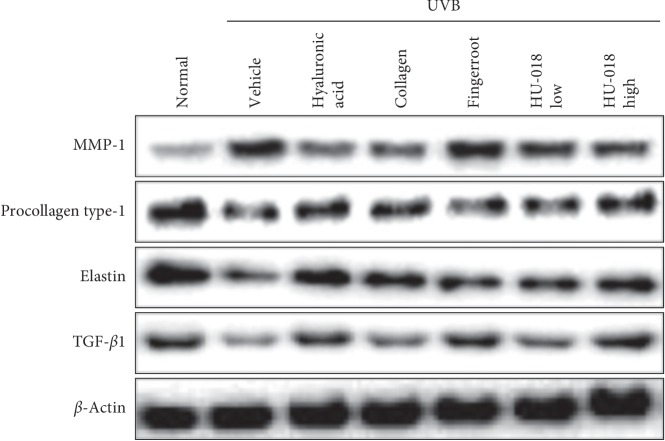
Protein expression of MMP-1, procollagen type I, elastin, and TGF-*β*1 in UVB-irradiated hairless mice quantified by western blot analysis. MMP, matrix metalloproteinase; UVB, ultraviolet B; TGF, transforming growth factor. Normal, non-irradiated group; vehicle, UVB-irradiated group.

To confirm the antiwrinkle effects of HU-018 treatment, we analyzed the protein levels of matrix metalloproteinase-1 (MMP-1), procollagen type-1, elastin, and TGF-*β*1 in skin tissue of UVB-irradiated mice. The expression of MMP-1 was markedly increased by UVB irradiation, and it was reduced by HU-018 treatment to a similar or better level as that observed in positive controls (Figure 6). The suppressed expression of procollagen type-1, elastin, and TGF-*β*1 by UVB irradiation was also restored by HU-018 treatment to a similar or better level as that observed in positive controls.

## 4. Discussion

Scale-up is generally achieved by increasing batch size, with practical and robust methods developed for establishing a large-scale production facility. During the scaling-up process, all factors that can influence the formulation of the active agent must be considered, ranging from basic factors (such as the boiling time and exposure duration) to the selection of commercial-grade materials that will be economically feasible to use. This process of modifying the compound for commercial use can also modify the risk profile of the agent, including toxicity and unwanted hazardous reactions. Therefore, the focus of our nonclinical evaluation of HU-018 was to bridge the gap between the discovery of a potent antiwrinkle agent in the laboratory to human clinical trials by developing a suitable procedure for production and testing, including setting the initial starting doses for clinical trials that balance antiaging effects with the level of risk for adverse effects (no-observed-adverse-effect level). The protective effects of HU-018 against UVB-induced skin damage described in our study are comparable to those in a previous study [6]. Therefore, we propose that HU-018 could be used in a clinical trial to evaluate its biological effects and the economic feasibility of production. We have confirmed the nontoxicity of HU-018 in a previous study in Sprague Dawley rats and beagles to establish that HU-018 met the requirements for commercialization. In addition, our previous study showed that HU-018 had a cytoprotective effect on HaCaT cells [7].

Photoaging is characterized by the formation of fine and coarse wrinkles, in combination with skin shallowness, histological changes, roughness, dryness, and various cutaneous changes, including altered skin barrier function [24]. Previous studies have described the effects of UVB radiation on epidermal morphology, such as increasing the SC thickness and the associated disruption of the permeability of the SC barrier, increases in TEWL and decreases in SC hydration [25, 26]. Additionally, a cornified cell envelope is composed of filaggrin, loricrin, and involucrin, and it contains differentiated epidermal keratinocytes and corneocytes. One of the most important roles of the cornified cell envelope is maintaining skin barrier function and moisture [27]. In this study, we found that HU-018 can reduce skin damage, decrease TEWL and EI, and increase the level of skin hydration in an animal model of photoaging.

The major changes in photoaged skin are localized to the dermal connective tissue, and they have been evaluated by histological and ultrastructural studies [28]. Morphological alteration of extrinsically aged skin is characterized by increased collagen degradation, sparse distribution of collagen fibers, accumulation of abnormal elastic tissue, presence of stellate phenotype of fibroblasts, thickening of the epidermis, and increased biosynthetic activity [29]. Repeated exposure to UV radiation also causes the accumulation of partially degraded collagen in the dermis, leading to wrinkles and skin damage, which are visible signs of photoaging [30]. Since skin roughness can be affected by thickened epidermis, epidermal thickness can be quantified to reflect skin damage due to UV irradiation using a histological feature of photoaged skin [31]. The increased epidermal thickness due to UV exposure can help protect the skin from further UV damage [32]. In this study, we performed H&E staining and Masson's trichrome staining to evaluate the histological alternations in the dorsal skin upon UVB irradiation. The results demonstrated that HU-018 administration reduced the epidermal thickening after UVB exposure, underlining the protective effects of HU-018.

HA exists at the periphery and interfaces of elastin fibers and collagen, and it can help hold elastin and collagen to maintain a proper configuration. However, these connections with HA are particularly decreased in aged skin, and might lead to the presence of fine lines, wrinkles, and nasolabial folds in the skin due to the disorganization of collagen and elastin fibers [33]. HA is a key molecule involved in skin moisture and helps maintain healthy skin by preventing aging and retaining skin hydration [16]. The synthesis of HA has been reported to be increased by growth factors such as TGF-*β* and synthesized from Has in the inner surface of the cell membrane [34]. Levels of hyaluronan synthase 2 (Has2), an enzyme that generates HA which is involved in moisturization, are reduced by UV irradiation [35]. Additionally, elastin is a well-understood connective tissue protein that is initially synthesized from the precursor molecule tropoelastin [36]. The microarray expression analysis performed in this study revealed that many skin hydration-related genes were also expressed in the HU-018 administration group. Our results showed that HU-018 at a high dose upregulated Has2, TGF-*β*3, and elastin gene expression in the UVB-irradiated group. This was accomplished by enhancing collagen synthesis, including an increased expression of TGF-*β*1, procollagen type-1, and elastin.

## 5. Conclusions

HU-018 treatment promoted skin hydration processes in a UVB-irradiated hairless mouse model by modulating elastin, involucrin, filaggrin, and loricrin expression. This was accomplished through enhanced collagen synthesis, including an increased expression of TGF-*β*1, procollagen type-1, and elastin. Based on these findings, we propose that HU-018 may be a good skin hydration agent for skin care.

## Data Availability

The data used to support the findings of this study are available from the corresponding author upon request.

## Supplementary Materials

Supplementary Materials Supplementary Figure 1: Effects of HU-018 on involucrin, filaggrin, and loricrin expression in UVB-irradiated HaCaT cells. Expression of (a) involucrin, (b) filaggrin, and (c) loricrin mRNA was determined by qRT-PCR. ^##^*p* < 0.01 and ^###^*p* < 0.001 versus the UVB-irradiated vehicle group. Nor, non-irradiated group; veh, UVB-irradiated group.
