# Supplementary material for: Skin Hydration Effects of Scale-Up Fermented Cyclopia intermedia against Ultraviolet B-Induced Damage in Keratinocyte Cells and Hairless Mice
Source: Evid Based Complement Alternat Med. 2020 Jan 11;2020:3121936. doi: 10.1155/2020/3121936 (PMC6982363; doi:10.1155/2020/3121936)
Supplement: Supplementary Materials — Supplementary Figure 1: Effects of HU-018 on involucrin, filaggrin, and loricrin expression in UVB-irradiated HaCaT cells. Expression of (a) involucrin, (b) filaggrin, and (c) loricrin mRNA was determined by qRT-PCR. ##p < 0.01 and ###p < 0.001 versus the UVB-irradiated vehicle group. Nor, non-irradiated group; veh, UVB-irradiated group. [file 3121936.f1.docx]

**
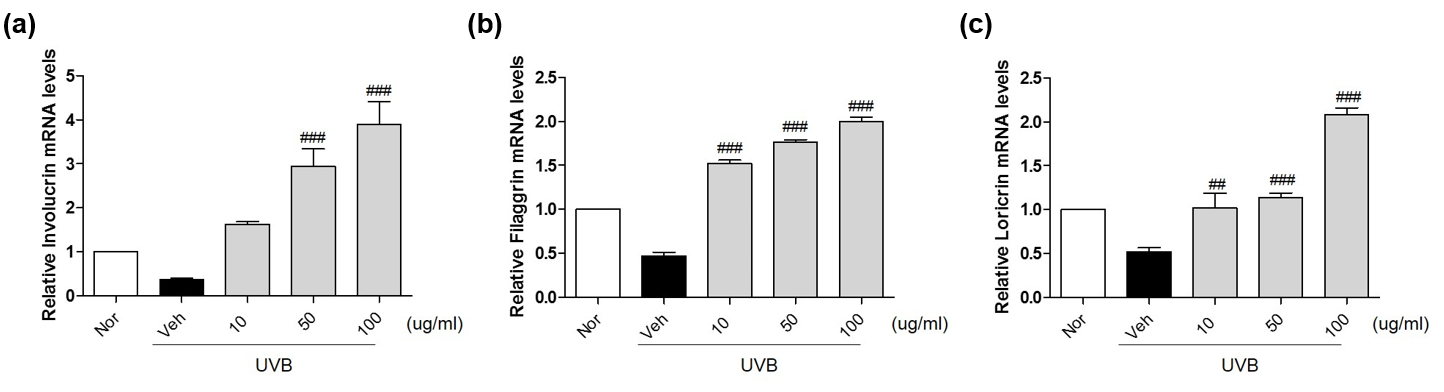
**

Supplementary Figure 1. Effects of HU-018 on involucrin, filaggrin, and loricrin expression in UVB-irradiated HaCaT cells. Expression of (a) involucrin, (b) filaggrin, and (c) loricrin mRNA was determined by qRT-PCR. ##*p* < 0.01 and ###*p* < 0.001 versus the UVB-irradiated vehicle group. Nor, non-irradiated group; vehicle, UVB-irradiated group.
